# Reports on the Hospitals of the United Kingdom

**Published:** 1911-12-16

**Authors:** Henry Burdett


### REPORTS ON THE Hospitals of the United Kingdom.

By SIR HENRY BURDETT, K.C.B., K.C.V.O.

### LVIII.—The Alexandra Bromo-Iodine Hospital, Woodhall Spa.

This institution was founded in 1890 and is fulfilling the objects of its founders, which were to provide especially for "patients suffering from rheumatoid arthritis, the sequelæ of acute rheumatism, neurasthenia, gouty arthritis, and complaints associated with the uric acid diathesis, eczema, psoriasis, and the complaints peculiar to women." The treatment consists of various kinds of baths, massage, the serum treatment, etc., under the superintendence of the honorary medical officers. Reports of these gentlemen claim that experience proves this treatment to be greatly beneficial in the case of from 40 to 45 per cent. of the patients who come under their care. The patients come from twenty-four English counties, and a few from Ireland.

We inspected this hospital with genuine pleasure because it exhibited evidence throughout of the care and devotion of the nurses and resident staff, which must make the Alexandra Hospital Spa a place which patients are likely to leave with regret and to reside in with pleasure. Nothing that good management, forethought, and wise administration can secure seems omitted, except perhaps, what is too usual in convalescent homes, that the day room space for women especially is too restricted, so that the chairs have to be crowded round the rooms close together, when more floor space would add to the hygienic efficiency of the rooms and contribute to the reasonable comfort of their occupants. This institution is fortunate in having for its Matron Miss A. M. Elliot, who was trained at the London Hospital, and has been a member of the Royal National Pension Fund for Nurses for some nineteen years. Miss Elliot seems to be able to give the maximum of satisfaction to everybody connected with the hospital, which she maintains in a high state of efficiency, whilst she is able to enforce a wise economy in every department of the management. This hospital owes its origin largely, if not entirely, to one man, an energetic vicar of Blankney, the Rev. J. O. Stephens, who is evidently a remarkable man in many ways, for he has succeeded in providing a singularly well-equipped and excellent building, for the relief of a type of sufferers who deserve our sympathy to an extent which is probably not surpassed by those afflicted with any other disease. Mr. Stephens has also succeeded in awakening a true spirit of devotion to the cause of the sick in the case of everybody charged with the duty of attending the patients, and making the working of the Alexandra Hospital as economical and efficient as possible. Another liberal benefactor who is entitled to public recognition is Mrs. Weigall, late Baroness von Eckardstein, a daughter of the late Sir Blundell Maple.

A striking tablet adorns this hospital bearing the following inscription: "This Hospital was built A.D. 1890 by the efforts of the Reverend J. O. Stephens, Rector of Blankney, and was the object of his unremitting care and supervision until he left the diocese July 1903

LAUS DEO."

